# Study on Behavior of Steel Hoop Connections for Raw Bamboo Members

**DOI:** 10.3390/ma14237253

**Published:** 2021-11-27

**Authors:** Chao Hu, Rui Cheng, Qilin Cheng, Jichun Liu

**Affiliations:** 1 Key Laboratory of New Technology for Construction of Cities in Mountain Area (Ministry of Education), Chongqing University, Chongqing 400045, China; huchao@cqu.edu.cn (C.H.); zuzudd@163.com (Q.C.); liujispring520@163.com (J.L.); 2 School of Civil Engineering, Chongqing University, Chongqing 400045, China

**Keywords:** steel hoop, raw bamboo, frictional properties, finite element analysis, design formula, reliability index

## Abstract

Bamboo structures have various types of connections, such as bolting and lashing. One crucial issue in bamboo structures is that the connection with bolts and nails has a lower load-carrying capacity associated with the bamboo failure resulting from the bolt or nail invading them. This paper focuses on the connection for raw bamboo members with steel hoops (BHC), of which the two semi-circular steel hoops are fastened to the raw bamboo with high-strength bolts. The sliding friction is controlled by the interfacial pressure, which can be increased by tightening the bolts. A push-out experiment on thirty-six specimens was conducted considering the following two parameters: the different surface conditions of raw bamboo (with or without the epidermis) and the different interfacial pressure. The test results mainly showed the two failure modes of specimens under certain conditions: continuous longitudinal slip after the vertical load reached the peak; and the steel hoop stuck in the bamboo skin after a period of slip. It is found that the sliding friction was controlled by the interfacial pressure, and the difference in the anti-sliding capacity between the epidermal bamboo specimen and the non-epidermal bamboo specimen was magnified with the increase of interfacial pressure. The contact stress on the surface of bamboo is approximately uniformly distributed based on the finite element analyses. The interfacial pressure can be predicted by the torque value of the digital electronic torque wrench and the equations established by mechanical analysis, respectively. Moreover, the design formulae of bearing capacity for BHC under three guaranteed rates (50%, 95%, and 99.9%) were developed based on probability theory, while the fourth design formula was derived by regression analysis. The reliability indices of the four design formulae were up to 0.07, 1.44, 3.09, and 0.97, respectively, and the resistance partial coefficients were suggested accordingly.

## 1. Introduction

Bamboo is an environmentally-friendly building material with excellent mechanical properties, especially tensile and compressive strength [1]. However, the connections are the difficult parts of bamboo construction.

**Figure 1:**
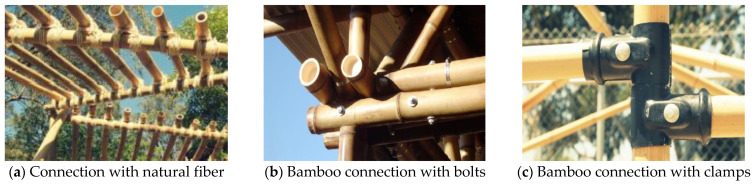
Types of bamboo connections [4] Open Access.

For the bamboo structure, there are many types of connections [2,3,4,5]. Traditionally, tying with natural fiber was the most common approach to connecting the bamboo members, as shown in Figure 1a. However, the tying tightness is greatly influenced by the tying skill, which may affect the behavior of connections. Therefore, bolts were introduced in bamboo connections of bamboo structures (Figure 1b,c) [6,7,8,9,10], which change the way the bamboo structure is connected. They are widely used in modern construction by employing modern tool electric drill machines. However, it possesses the following disadvantages: (1) The bearing capacity of the raw bamboo is weakened due to the drilling and cutting of raw bamboo; (2) The connections need to be reinforced by grouting, outer hoop, and other reinforcement, which increase fabrication difficulty and cost; (3) Because the raw bamboo needs grouting inside in some cases, the installation and disassembly are difficult and it cannot be reassembled after disassembly.

**Figure 2:**
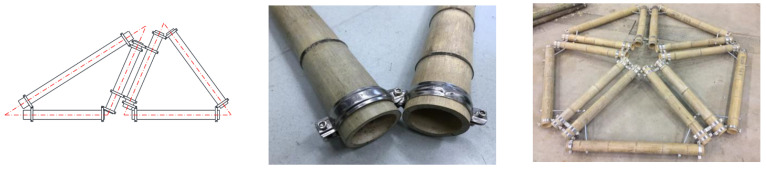
The bamboo connection with steel hoops (BHC).

The connection for raw bamboo culms wrapped with steel hoops was proposed recently and has been attracting a lot of interest among researchers [11,12,13], as shown in Figure 2. This type of connection has the following features: (1) The sliding friction is controlled by the interfacial pressure, which can be predicted by the torque value of the digital electronic torque wrench and the equations established by mechanical analysis, respectively. The interfacial pressure is improved by tightening the bolts; (2) It is easy for installation and disassembly; (3) This type of bamboo connection can be used in the spatial structure of raw bamboo (Figure 2), which can enhance the integral behavior of the bamboo structure. Richard et al. [11] propose three new beam–column bamboo connections capable of transmitting moment. Each joint is comprised of five thin steel clamps tightened around the culms. The study demonstrated that clamp moment connections are a feasible alternative to improve the structural performance and versatility of bamboo structures. The bamboo culms wrapped with steel rings under axial and transverse loading were tested by some researchers [12,13]. It was found that axial experiments showed maximum loads in the range 7.5–18 kN with signs of high plastic deformations that usually begin at about 5 kN. The failure of specimens subjected to transverse loads showed ductile characteristics.

The frictional properties of the connection for raw bamboo members wrapped with steel hoops (BHC) have a significant influence on the behaviors of the joint. However, the frictional properties are not clear. It is of great interest to investigate the frictional properties of BHC. The frictional properties of the composite interface between bamboo and steel hoops (BHC) were studied in this paper, considering the different surface conditions of raw bamboo (with or without the epidermis) and the different interfacial pressure. The purposes of this study were to investigate the possible failure modes, to find the proper method for calculating the sliding friction force, and to provide reasonable design details for the BHC.

## 2. Experimental Program

### 2.1. Specimen Design

Thirty-six push-out specimens were designed and divided into three groups, namely PO-1-series specimens under the same contact pressure, PO-2-series specimens under different contact pressures, and PO-3-series specimens without the epidermis under different contact pressures. The three groups of specimens have the following purpose, respectively: (1) Investigating the difference of friction force under the same contact pressure and developing the design formula of bearing capacity for BHC under different guaranteed rates based on probability theory; (2) Studying the difference of friction coefficients under the different contact pressure and developing the design formula by regression analysis; (3) Investigating the effect on the frictional properties of the different surface conditions (raw bamboo with or without the epidermis) and developing the design formula for raw bamboo without the epidermis.

**Figure 3:**
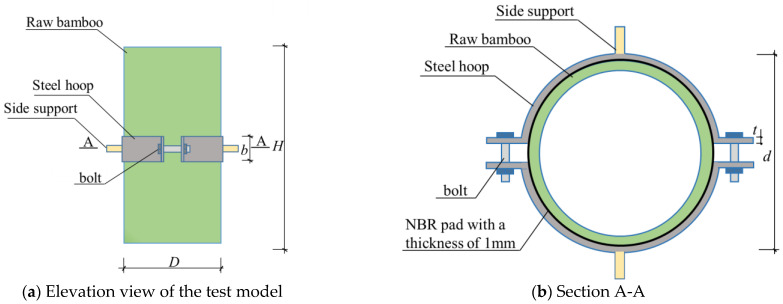
Dimensions of specimens.

As load-bearing bamboos, the bamboo age should not be less than 4 years, and the moisture content is generally between 8% and 12% [14]. The raw bamboo used in this experiment is 4 years old and is produced in the Shunan Bamboo Sea, Yibin, Sichuan Province, China. The specimens have the following common details (Figure 3) as summarized in Table 1: the thickness of steel hoop *t* of 0.8 mm; diameter of steel hoop *d* of 110 mm; width of steel hoop *b* of 23 mm; diameter of bamboo *D* of 110 mm (standard deviation of 0.05); thickness of bamboo of 10.3 mm (standard deviation of 0.04); the height of bamboo *H* of 300 mm. The non-epidermal bamboos were prepared by scraping off their skin with a knife. All steel hoops in this study are finished products, which are cold-formed from thin plates with 23 mm width and 0.8 mm thickness. The middle area along the width direction of the plate is convex, and the convex width is 13.8 mm, that is, the actual contact width between the steel hoop and bamboo is 9.2 mm. Each semi-circular steel hoop (Figure 3) poses a curved portion, followed at each side by two sharp curves in the transition to the flat ends (lugs). In the lugs, holes are drilled that allow us to fasten the steel hoop around the bamboo using bolts of 6 mm diameter. The side support consists of a screw (length of 30 mm and diameter of 10 mm) and a screw cap with a rectangular section (longer and shorter side lengths of 22 mm and 15 mm, respectively), an inner diameter of 10 mm, and a length of 20 mm. A round screw cap with a length of 15 mm and an inner diameter of 10 mm was welded to the middle of the semi-circular clamp and then connected with the side support. Considering that the inner surface of the steel hoop is smooth and nitrile butadiene rubber (NBR) pads are often used to increase the interfacial friction force in practical engineering, the NBR pad of 1-mm thick was attached to the inner side of the steel hoop, as shown in Figure 3. The two semi-circular steel hoops were then fastened to the raw bamboo by tightening the high-strength bolts with a digital electronic torque wrench. The connection (BHC) consists of the raw bamboo, the steel hoop, and the NBR pad. Therefore, the friction coefficients of steel hoop connections for raw bamboo members are specific to the surface of the bamboo and the NBR pad.

### 2.2. Material Properties

According to the recommendation of Chinese code GB/T 228.1-2010 [15], three tensile tests were conducted to measure the mechanical properties of steel. The specimens for raw bamboo were prepared and tested in accordance with JG/T 199-2007 [16] to determine the material properties. The material properties are summarized in Table 2, where *f*_y_, *E*_s_ and *f*_u_ are the yield strength of steel, the steel elastic modulus and the ultimate strength of steel, respectively.

### 2.3. Test Set-Up and Measuring Scheme

**Figure 4:**
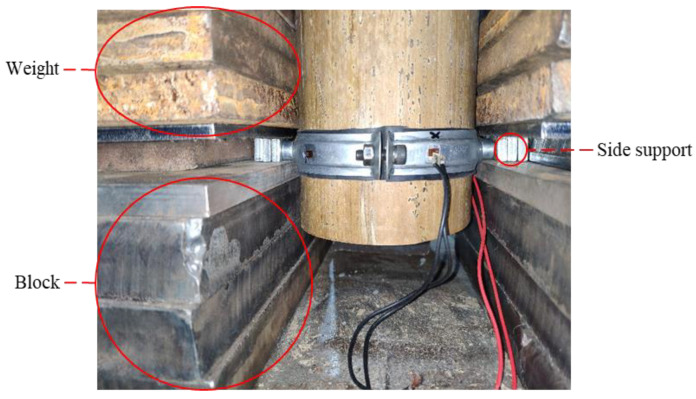
Fixation of side supports.

**Figure 5:**
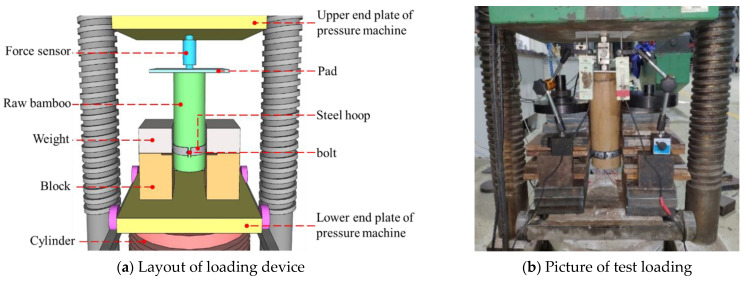
Loading device.

**Figure 6:**
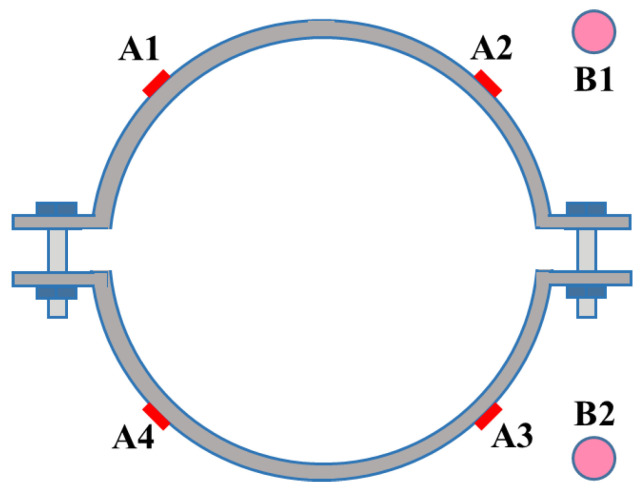
The layout of measuring points.

The steel hoop was installed horizontally on the bamboo tube, and two side supports were placed on the pier and fixed with heavy objects, as shown in Figure 4. The top end of the specimens was loaded vertically using a pressure testing machine. The applied force was recorded through a 10 kN force sensor (Figure 5). Moreover, a hybrid load-displacement controlled scheme of loading was applied. The load-controlled mode was adopted at a rate of 0.05 kN/min during the initial stage of loading. After the load reached the peak, the loading scheme was shifted to a displacement-controlled mode with a rate of 1 mm/min. The test stopped until the specimen slid significantly. As illustrated in Figure 6, two linear variable differential transducers (LVDTs), B1 and B2, were used to record the longitudinal slip of the specimens and four strain gauges A1~A4 were evenly attached around the steel hoop to measure the hoop strain. The high-strength bolts were tightened with a torque wrench. The interfacial pressure can be calculated according to the torque value and hoop strain, respectively.

## 3. Test Results

### 3.1. Load-Displacement Curves

**Figure 7:**
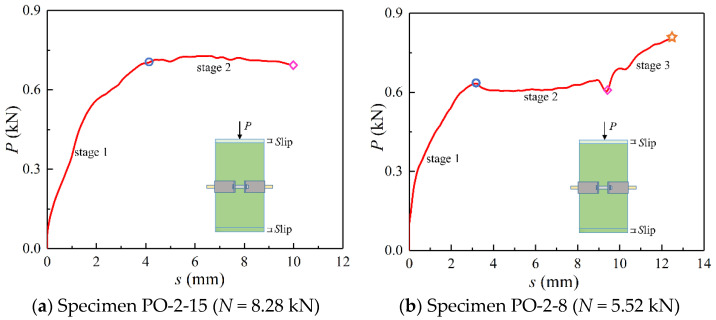
Different stages of the whole loading process of specimens.

Comparing the whole loading process of specimens, it could be divided into two categories, which correspond to two failure modes, respectively. Typical specimens are selected from the two categories of specimens for description and analysis, as shown in Figure 7. The whole loading process of all specimens included the load-rising stage (stage 1) and the sliding stage (stage 2). During the load-rising stage, the load increased linearly with the increase of displacement due to the compression of bamboo and the small gap between the connected loading devices, between the loading devices and the specimen, as well as between the measuring apparatus and the specimen. After the load reached the peak load *P*_u_, the specimens shifted to the sliding stage (stage 2), in which the load no longer increased or began to decrease, while the displacement kept increasing, indicating that the bamboo tube began to slip. As can be seen from Figure 7b, a few specimens such as specimens PO-2-8, PO-2-12, and PO-3-7 shifted to the second load-rising stage (stage 3) after a period of slip, resulting from the steel hoop stuck in the raw bamboo skin.

**Figure 8:**
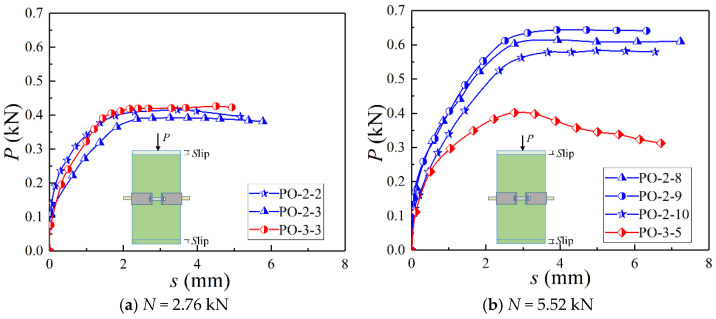
Load-displacement curves of specimens.

**Figure 9:**
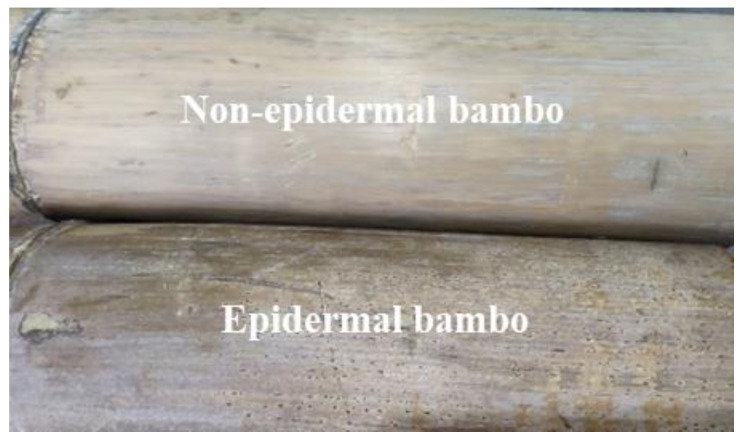
Two kinds of specimens.

Figure 8 demonstrates the load-displacement curves of specimens. It is found that, in the situation of the small contact pressure on the interface, the difference of bearing capacity between the epidermal bamboo specimen and the non-epidermal bamboo specimen was small. The difference gradually increased with the increase of the contact pressure. Since the surface of the raw bamboo without the epidermis was smooth and flat, it was in more contact with the steel hoop, while the surface of the raw bamboo with the epidermis was rough and uneven (higher friction coefficient) but in less contact with the steel hoop (Figure 9). In this case, the difference between the two kinds of specimens was quite small. Afterwards, the difference increased more with the steel hoop gradually in contact with the epidermal bamboo.

### 3.2. Deformation Capacity

**Figure 10:**
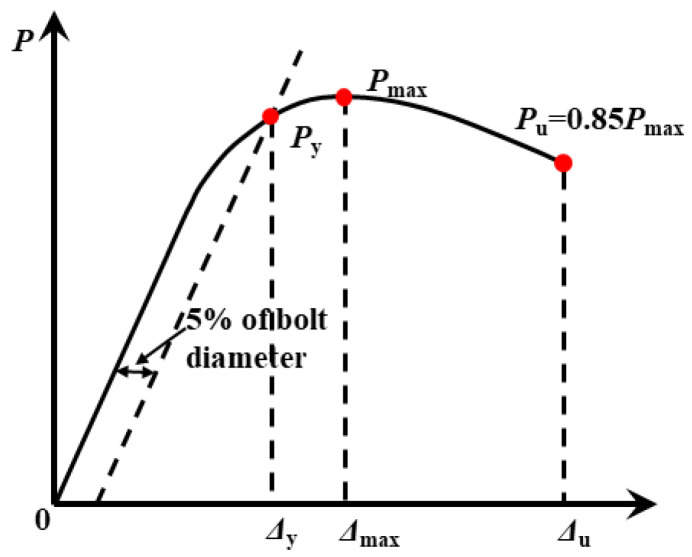
Determination of yield and ultimate point.

The yield point is specified in ASTM D5764-97a [17]. Based on Johansen’s yielding model, the 5% *d* (*d* is the bolt diameter) offset method is used to predict the yielding capacity and the stiffness of the connection, as shown in Figure 10. The ultimate point is interpreted as the point when the load dropped to 85% of the peak load or when the experiment is ended (the load does not drop below 85% of its peak load). The ductility coefficient calculated by Δ_u_/Δ_y_ is used to evaluate the deformability of the connections, where Δ_u_ and Δ_y_ are the ultimate and yield displacement, respectively.

**Figure 11:**
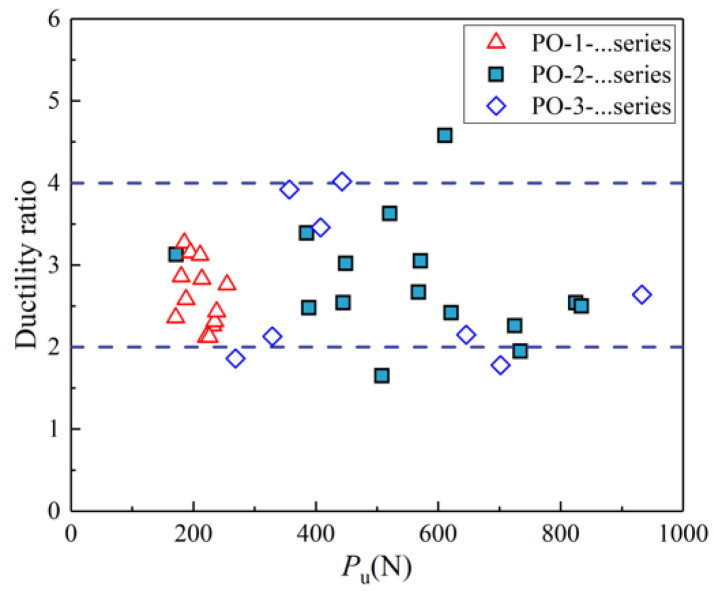
Comparison of ductility coefficients.

The ductility coefficients and stiffness of all the groups in this study are provided in Table 1. The stiffness ranges from 0.13 kN/mm to 0.28 kN/mm with a standard deviation of 0.043. It is found from Figure 11 that the values of ductility coefficients range from 1.65 to 4.58, and there is no obvious regular pattern among the three groups, resulting from the different test ending times of the specimens. According to the classification of the ductility coefficient presented by Bruhl et al. [18], the connection is classified as brittle, low ductile, moderate ductile, and high ductile, which correspond to the ductility coefficient values of <2, 2–4, 4–6 and >6, respectively. The values of the ductility coefficients of specimens are mostly greater than 2. Therefore, the connection for raw bamboo culms wrapped with steel hoops may be classified as low ductility.

### 3.3. Failure Modes

**Figure 12:**
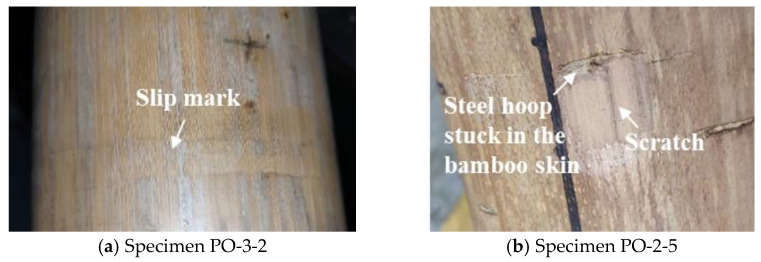
Failure modes of specimens.

The experimental results mainly showed two failure modes of all specimens: (1) During the initial stage of loading, there was no obvious phenomenon in the specimens and the axial displacement was small. When the specimens reached the peak load *P*_u_, obvious slip occurred in BHC, as shown in Figure 12a; (2) After a period of slipping, such as in specimens PO-2-5, PO-2-12, and PO-3-7, the bearing capacity rises again (the steel hoop stuck in the bamboo skin) resulting from the rough surface and heterogeneous diameters of bamboo tubes, as shown in Figure 12b.

Overall, the failure of the specimens in this study showed a certain ductility. Although these are some imperfections of the bamboo material, it is favorable for improving the bearing capacity and structural safety. The impact of this factor was not taken into account in this paper.

## 4. Finite Element Analyses

### 4.1. Finite Element (FE) Model

**Figure 13:**
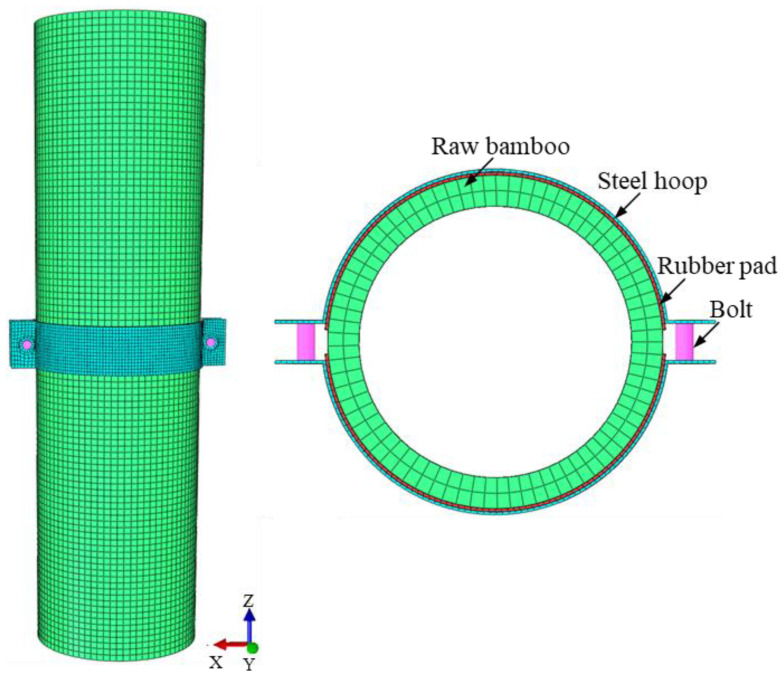
Finite element model.

The software ABAQUS [19] was used to simulate the bamboo connection with steel hoops subjected to the different pretension forces of high-strength bolts and the finite element (FE) model is shown in Figure 13.

The steel constitution employs the bi-linear stress–strain relation with the hardening modulus equal to 1% of the elastic modulus [20]. The elastic modulus and Poisson’s ratio of steel are 198 GPa and 0.29, respectively.

The material type of bamboo is anisotropic. Bamboo has different strengths and moduli of elasticity along and across grains for compression and tension. The axes x, y, and z were oriented along the radial, circumferential and axial directions, respectively. A transversely isotropic and functionally graded material was defined for the bamboo, with *E*_x_ and *E*_y_ varying linearly from 686 MPa at the inner edge, to 1611 MPa at the outer edge [21]. To account for the variation of the material properties through the culm wall thickness, an artificial radial temperature field with the function of *E*_x_ and *E*_y_ was prescribed [22]. The shear modulus in the plane x-y of isotropy can be determined by:

(1) $$G_{\mathrm{xy}}=\frac{E_{x}}{2\left(1+V_{\mathrm{xy}}\right)},$$

where *V*_xy_ is assumed as 0.22 [21].

The other material properties were assumed to be constant with position as: *E*_z_ = 12,000 MPa, *G*_xz_ = *G*_yz_ = 800 MPa, *V*_xz_ = *V*_yz_ = 0.01 [21].

Non-linear geometric effects were included in the analysis.

The material type of rubber is hyperelastic. There are several models of hyperelastic materials, where the Mooney–Rivlin model is the most common method [23,24]. It can predict the behavior of isotropic materials (e.g., rubber) with good accuracy.

The form of the deformation potential energy of the Mooney–Rivlin model is determined as:

(2) $$W=C_{10}(I_{1}-3)+C_{01}(I_{2}-3)+\frac{1}{D_{1}}{\left(J-1\right)}^{2},$$

where *C*_10_, *C*_01_, and *D*_1_ are specific parameters depending on temperature; *I*_1_ and *I*_2_ are the invariants of the deformation matrix deviator; *J* is the ratio of rubber volume after deformation to that before deformation.

Assuming rubber is regarded as the incompressible hyperelastic materials, the form of the deformation potential energy of the Mooney–Rivlin model can be expressed as:

(3) $$W=C_{10}(I_{1}-3)+C_{01}(I_{2}-3),$$

where *C*_10_ and *C*_01_ are 2.767 and 1.439 [24], respectively.

Surface-to-surface contact interaction with a hard contact property in the normal direction and a penalty function (the friction coefficient calculated by Equation (19)) in the tangential direction is applied to simulate the interaction between the bamboo and the rubber pad and the interaction between the rubber pad and the steel hoop. The MPC constraint is used to simulate the connection of bolting between the steel hoops and the bolts. The finite element (FE) model is presented in Figure 13. The different interfacial pressure is achieved by applying different bolt loads.

A solid element (C3D8R) is used for the bamboo and the steel hoop. A beam element is adopted for the bolt. The rubber pad is modeled using an eight-node hybrid solid element with reduced integration (C3D8H). By comparing FE models of different element mesh sizes, it is found that the mesh size of the steel hoop and the rubber pad were selected as 2 mm and the mesh size of the bamboo and the bolt were selected as 1 mm and 4 mm, respectively, to provide reliable accuracy and appropriate calculation efficiency.

### 4.2. Verification of FE Model

**Figure 14:**
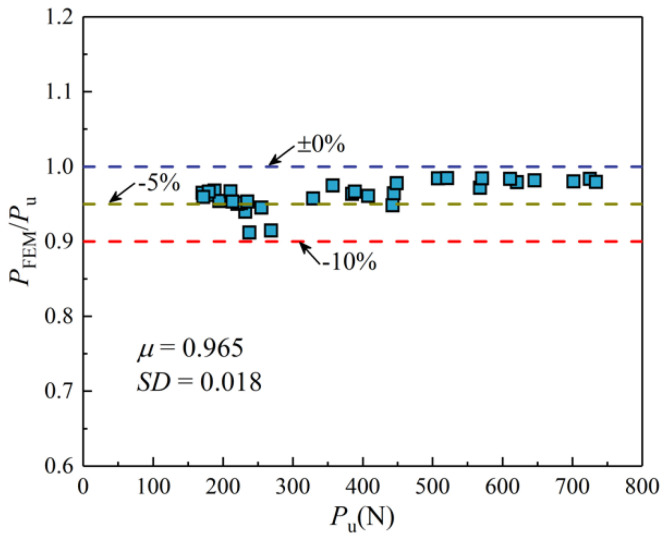
Comparison of ultimate bearing capacities for experimental results and FE results.

Table 1 and Figure 14 demonstrate the comparison of the bearing capacities for experiments and FE models. The mean value *μ* and standard deviation *SD* of the ratio *P*_FEM_/*P*_u_ are 0.965 and 0.018, respectively. As seen, the bearing capacities predicted by the FE models are lower than the test results. The deviation of the ratio *P*_FEM_/*P*_u_ is mostly within 5%, and with the increase of the bearing capacity, the deviation of *P*_FEM_/*P*_u_ is reduced, indicating that the simulated values are in good agreement with the experimental ones.

### 4.3. Contact Stress Distribution Analyses

To investigate the effects of the pretension force of high-strength bolts, noncircular cross-section of bamboo, and rubber pad on the contact stress distribution, the contact stress distribution analyses were carried out based on the FE model. The connection BHC with different pretension force *P*_c_ (0–2 kN), ellipticity *η* of bamboo (1.00, 0.95, and 0.90), and surface conditions of BHC (with or without the rubber pad) are simulated. Ellipticity *η* is a ratio of the semiminor axis of the ellipse of polarization to the semimajor axis.

**Figure 15:**
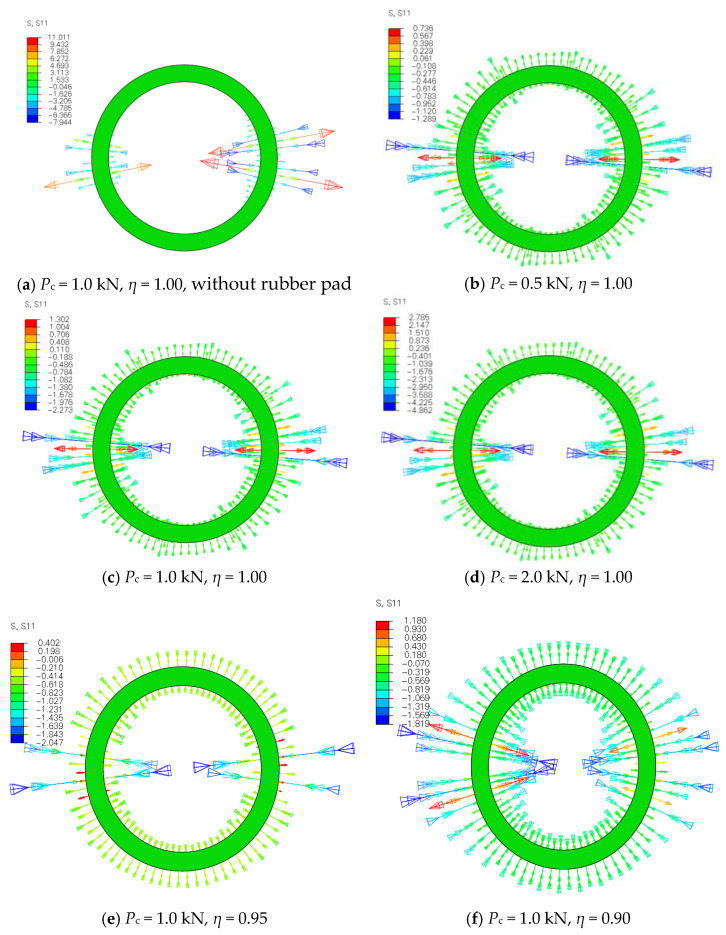
The contact stress distribution on the surface of bamboo.

Figure 15 demonstrates the contact stress distribution on the surface of the bamboo, where S11 represents the contact stress on the surface of bamboo. In the stress cloud figures, the boundary lines between different colors are the contours. As can be seen from Figure 15a, for the connection BHC without a rubber pad, the contact stress cannot be transferred to the middle region. It can be seen in Figure 15b–d that the region where the bolt load is applied is highly stressed, while the contact stress on the other region is approximately uniformly distributed. As the pretension force of high-strength bolts increases from 0 kN to 2 kN, the variation of the contact stress distribution is small. It can be seen from Figure 15c,e,f that, as ellipticity *η* decreases from 1.00 to 0.90, the contact stress gradually decreases from both ends to the middle region, indicating that the contact stress gradually does not obey the uniform distribution with the decrease of ellipticity *η* of bamboo. The circular cross-section of bamboo is suggested in this paper. Therefore, the contact stress can be regarded as a uniform distribution.

## 5. Working Mechanism of the Clamp

The contact pressure of BHC was applied by tightening the bolts. Once a vertical load was applied to the raw bamboo, the BHC generated friction, which was balanced with the load.

### 5.1. Determination of the Interfacial Friction Force

The vertical load was balanced with the interfacial friction force *F*_f_. Thus, the equation is expressed as:

(4) $$F_{f}=P_{u},$$

where *P*_u_ is the peak load obtained by the experiment.

### 5.2. Relationship of the Contact Stress and the Circumferential Tensile Force

As mentioned in Section 4.3, the contact stress (*n*) can be regarded as a uniform distribution. The contact stress (*n*) is determined by:

(5) $$n=\frac{N}{S}=\frac{N}{2\pi bR},$$

where *S* is the surface area of the steel hoop, *R* and *b* is the radius and width of the steel hoop, respectively.

**Figure 16:**
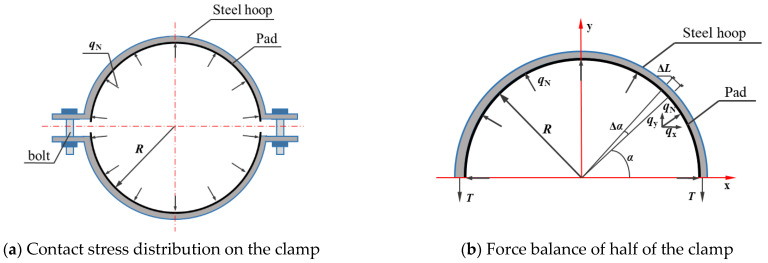
Mechanical analysis diagram of the clamp.

The unit width of the clamp is 1 mm, and the linear load *q*_N_ of the per-unit width of the clamp is shown in Figure 16a. The contact pressure is in equilibrium with the circumferential tensile force *T* in accordance with the mechanical analysis of half of the clamp, as shown in Figure 16b.

(6) $$q_{N}=n\times 1=\frac{N}{2\pi bR}.$$

The component of the linear load *q*_N_ in the *y*-direction can be expressed as:

(7) $$q_{y}=q_{N}\sin\alpha .$$

The differential variable d*N*_y_ of the contact pressure is calculated by:

(8) $$dN_{y}=q_{y}bdl=(q_{N}b\sin\alpha )Rd\alpha =q_{N}bR\sin\alpha d\alpha .$$

According to the force balance of the half clamp in the vertical direction (see Figure 16b), Equation (9) is developed to predict the circumferential tensile force *T*:

$$\sum F_{y}=0,2T-\int_{0}^{\pi }dN_{y}=0$$

(9) $$T=\frac{bR}{2}\int_{0}^{\pi }q_{N}\sin\alpha d\alpha =q_{N}bR.$$

Substituting Equation (6) into Equation (9), the relational equation between the contact pressure *N* and the circumferential tensile force *T* can be expressed as:

(10) $$T=q_{N}bR=\frac{N}{2\pi bR}bR=\frac{N}{2\pi }.$$

### 5.3. Determination of the Circumferential Tensile Force

#### 5.3.1. Relationship of the Circumferential Tensile Force and the Final Tightening Torque

Equation (11) is derived in accordance with GB 50205-2020 [25] to calculate the pretension force *P*_c_ of a high-strength bolt:

(11) $$P_{c}=\frac{T_{c}}{K\cdot d},$$

where *T*_c_ is the final tightening torque of a high-strength bolt, *K* is the torque coefficient and *d* is the diameter of the high-strength bolt.

The pretension force *P*_c_ of the bolt is equal to the circumferential tensile force *T*. Thus, the equation is expressed as:

(12) $$T=P_{c}=\frac{T_{c}}{K\cdot d}.$$

#### 5.3.2. Relationship of the Circumferential Tensile Force and the Circumferential Strain

Contact stress *σ*_θ_ on the section of the clamp can be calculated by:

(13) $$\sigma_{\theta }=\frac{T}{tb},$$

where *t* is the thickness of the steel hoop.

Radial stress *σ*_r_ inside the clamp is determined as follows:

(14) $$\sigma_{r}=-q_{N}=-\frac{T}{bR}.$$

On the basis of the generalized Hooke’s law, the following equation is derived to predict the circumferential strain of steel hoop *ε*:

(15) $$\begin{array}{cc} \varepsilon & =\frac{1}{E}(\sigma_{\theta }-v\sigma_{r})=\frac{1}{E}\left[\frac{T}{tb}-v\left(-\frac{T}{bR}\right)\right]\\ & =\frac{T}{bE}\left(\frac{1}{t}+\frac{v}{R}\right) \end{array}$$

Therefore, the circumferential tensile force *T* can be expressed as:

(16) $$T=\frac{\varepsilon tbRE}{R+vt},$$

where *ν* is the Poisson’s ratio of steel and *E* is the elastic modulus of steel.

### 5.4. Determination of the Contact Pressure

#### 5.4.1. Relationship of the Contact Pressure and the Final Tightening Torque

According to Equations (10) and (12), the contact pressure *N* can be expressed as:

(17) $$N=\frac{2\pi T_{c}}{K\cdot d}.$$

#### 5.4.2. Relationship of the Contact Pressure and the Circumferential Strain ε

On the basis of Equations (10) and (16), the contact pressure *N* is predicted by:

(18) $$N=\frac{2\pi t\varepsilon bER}{R+t\nu }\approx 2\pi t\varepsilon bE\left(R\gg t\nu \right).$$

### 5.5. Determination of the Friction Coefficient

Equation (11) is derived according to Equations (4) and (17) to determine the friction coefficient *μ*:

(19) $$\mu =\frac{F_{f}}{N}=\frac{PKd}{2\pi T_{c}}.$$

In accordance with Equations (4) and (18), the friction coefficient *μ* can be expressed as:

(20) $$\mu =\frac{F_{f}}{N}=\frac{P}{2\pi t\varepsilon bE}.$$

## 6. Design Formula for the Interfacial Friction Force

When the bolt load is applied, the surface of bamboo at the end of the clamp suffers higher stress resulting from the traction force. When the stress in this area reaches the maximum contact stress (compression strength perpendicular to grain), the maximum bolt adjustment is attained, and the connection for raw bamboo members wrapped with steel hoops reaches the maximum bearing capacity. The specimens for raw bamboo yielded a compression strength perpendicular to a grain of 27.62 Mpa [5]. Based on the FE analysis, when the maximum contact stress reaches the compression strength, the pretension force of bolt and is 11.36 kN. In practical engineering applications, the diameter and grade of bolts can then be determined according to the pretension force of bolt. The contact pressure can then be calculated by Equation (17). Since the interface friction coefficient has not been determined, the design methods of the interface friction coefficient need to be proposed to calculate the bearing capacity of the connection.

### 6.1. Design Formula under Different Guarantee Rates

The steel hoop is not in full contact with the bamboo due to the non-round section and the uneven surface and heterogeneous diameters of the bamboo. Considering the defects of raw bamboo, the series of PO-1 specimens are designed to investigate the difference of friction force under the same contact pressure.

**Figure 17:**
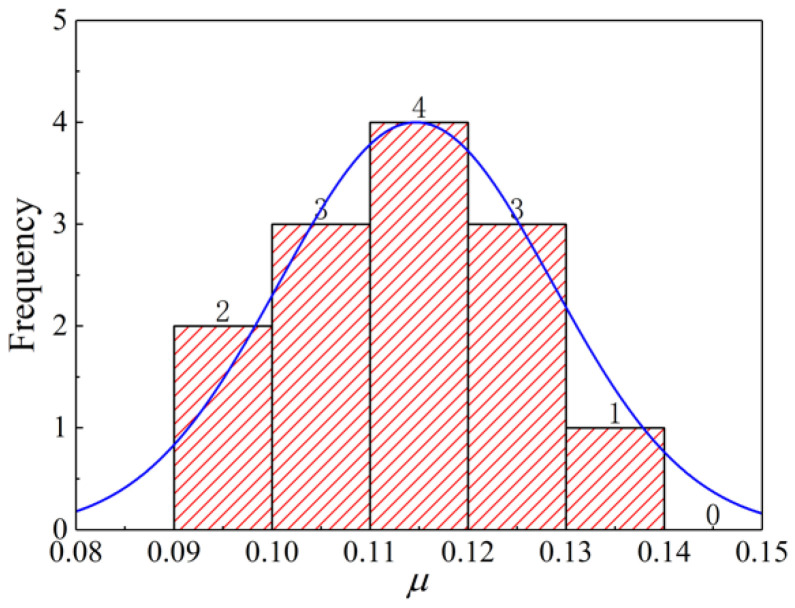
Histogram of the friction coefficient frequency *μ*.

The data of the interfacial friction coefficient *μ* of specimens PO-1-1~PO-1-13 obey a normal distribution according to the Shapiro–Wilk method [26,27,28]. Figure 17 also shows that the friction coefficient *μ* follows this rule.

**Figure 18:**
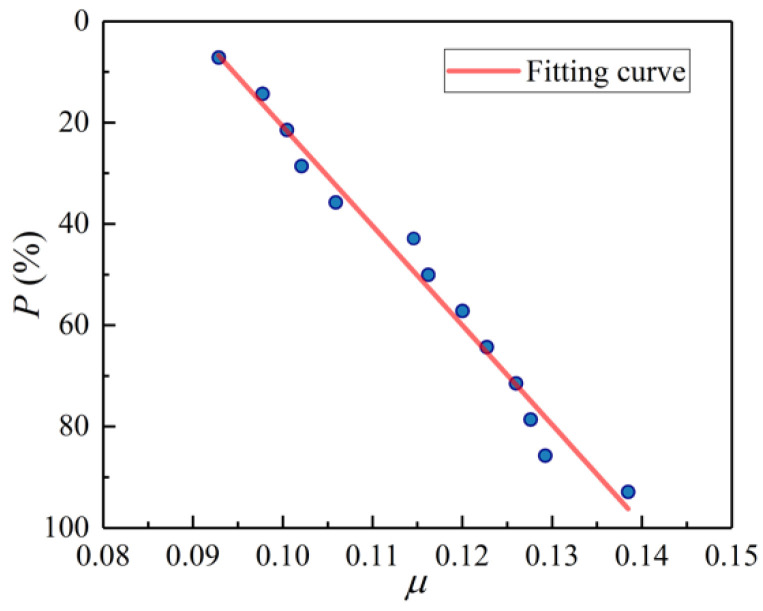
Distribution diagram of the interfacial friction coefficient *μ*.

Moreover, the interfacial friction coefficient *μ* is arranged in ascending order and is drawn on the normal probability graph paper [29], as shown in Figure 18, in which the average rank is used as the estimation of the failure probability *P*, and the guaranteed probability is *G*_t_ = 1 − *P*. It can be seen from Figure 18 that there is approximately a linear relationship between *μ* and *P* and the correlation coefficient calculated by the least square method is 0.9908. Combined with the test result of the Shapiro–Wilk method, it proves that the interfacial friction coefficient *μ* obeys a normal distribution.

By subtracting 0, 1.645, and 3.09 times standard deviation from the average value of the friction coefficient *μ*, the *μ* values are obtained corresponding to the guarantee rate *G*_t_ of 50%, 95%, and 99.9% [30,31,32]. Substituting them into Equation (19) respectively, the following equations are derived to predict the interfacial friction force *F*_f_ under different guarantee rates *G*_t_.

For the interfacial friction force *F*_f_ under guarantee rate *G*_t_ of 50%:

(21) $$F_{f}=0.115N.$$

For the interfacial friction force *F*_f_ under the guarantee rate *G*_t_ of 95%:

(22) $$F_{f}=0.096N.$$

For the interfacial friction force *F*_f_ under the guarantee rate *G*_t_ of 99.9%:

(23) $$F_{f}=0.077N.$$

**Figure 19:**
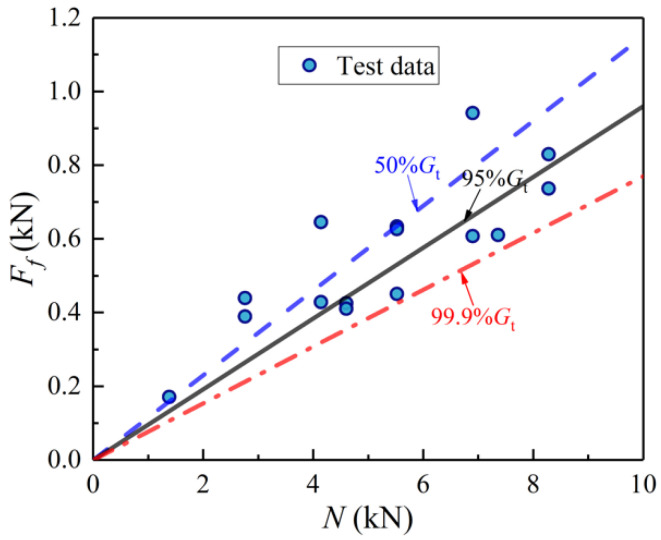
*F*_f_-*N* curves for different guaranteed rates.

Figure 19 demonstrates the *N*-*F*_f_ curves of BHC under 50% *G*_t_, 95% *G*_t_, and 99.9% *G*_t_, called *G*_t_-*F*_f_-*N* curves, which are a set of *F*_f_-*N* lines composed of different guarantee rates *G*_t_. The data of specimens PO-2-1~PO-2-15 were included in the figure. All test data points are above the 99.9% *G*_t_-*F*_f_-*N* curve and the 50% *G*_t_-*F*_f_-*N* curve is in the middle of the test data points. Overall, the different guaranteed rates obtained through probability analysis can reflect the security degree of the design formulae well.

### 6.2. Fitting Design Formula

#### 6.2.1. Raw Bamboo with Epidermis

**Figure 20:**
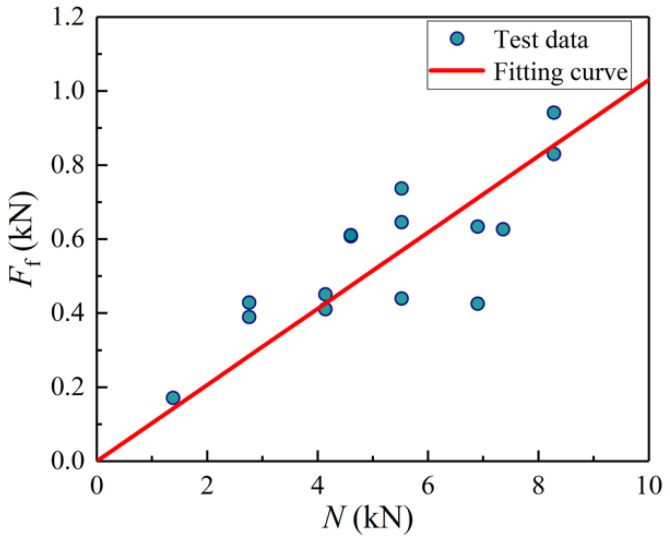
*F*_f_-*N* fitting curve.

Through regression analysis of the data of specimens PO-2-1~PO-2-15 (Figure 20), Equation (24) is derived to predict the interfacial friction force *F*_f_ for raw bamboo with the epidermis:

(24) $$F_{f}=0.103N.$$

Thus, the interfacial friction coefficient *μ* can be expressed as:

(25) $$\mu =\frac{F_{f}}{N}=0.103.$$

The friction coefficient *μ* of the fitting equation is 0.103 and the standard deviation compared with the test data is 0.152, which indicates that the fitting curve agrees well with the test data. Based on probability theory, the fitting formula is equivalent to the 81.94% *G*_t_-*F*_f_-*N* formula.

#### 6.2.2. Raw Bamboo without Epidermis

**Figure 21:**
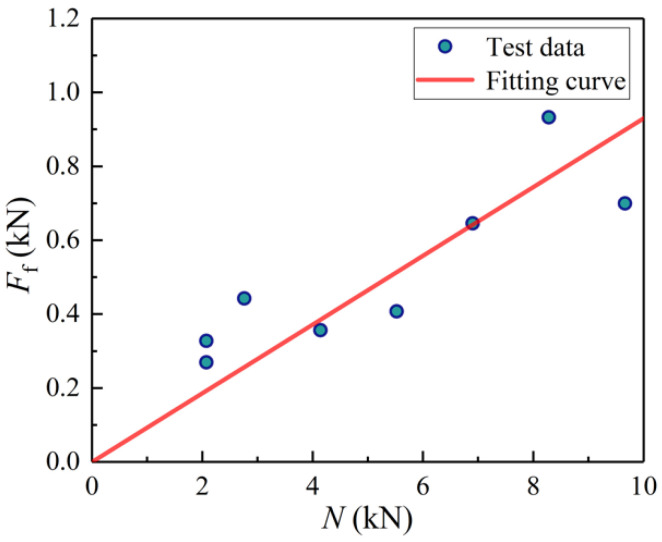
*F*_f_-*N* fitting curve for raw bamboo without the epidermis.

Through regression analysis of the data of specimens test pieces PO-3-1~PO-3-8, the *F*_f_-*N* curve for raw bamboo without the epidermis is shown in Figure 21. The following equation is obtained to calculate the interfacial friction force *F*_f_ for raw bamboo without the epidermis:

(26) $$F_{f}=0.093N.$$

Therefore, the interfacial friction coefficient *μ* can be expressed as:

(27) $$\mu =\frac{F_{f}}{N}=0.093.$$

The friction coefficient *μ* of the fitting equation is 0.093 and the standard deviation compared with the test data is 0.133. It indicates that the fitting curve agrees well with the test data. By comparing Equation (25) with Equation (27), it can be found that the epidermal raw bamboos show a larger interfacial friction force than non-epidermal ones.

### 6.3. Comparison of the Proposed Design Method and the Test Data of Other Connections

To compare the proposed design method with the test data of the other bamboo connection method, a total of 15 specimens were collected [5], as shown in Table 3. For the specimens of the WS-series, the Chinese fir and the steel plate were inserted into the inner cavity of the raw bamboo. The screw rod was then threaded through the raw bamboo, Chinese fir, and steel plate.

When the bolt load is applied, the surface of bamboo at the end of the clamp suffers higher stress resulting from the traction force. When the stress in this area reaches the maximum contact stress (compression strength perpendicular to grain), the maximum bolt adjustment is attained, and the connection for raw bamboo members wrapped with steel hoops reaches the maximum bearing capacity. The specimens for raw bamboo yielded a compression strength perpendicular to grain of 27.62 Mpa [5]. Based on the FE analysis, when the maximum contact stress reaches the compression strength, the pretension force of the bolt is 11.36 kN, and the bearing capacity *P*_uc_ calculated by Equations (19) and (23) is 5.52 kN. The bearing capacity *P*_uc_ is compared with the bearing capacities of the other bamboo connection method. The mean value of *P*_uc_/*P*_u_ is 0.481 with a range from 0.228 to 1.075. Although the bearing capacity of a single connection is mostly lower than that of the other bamboo connection, the advantage of the connection for raw bamboo members wrapped with steel hoops is that, in practical engineering applications, the number of the connections can be configured according to the design load.

## 7. Reliability Analysis

### 7.1. Reliable Index

The structural limit state equation [33,34] is expressed as:

(28) $$Z=R_{u}-R_{n}=0.$$

Then, the limit state equation for structural random variables becomes:

(29) $$Z=R_{u}-R_{n}=\mu \cdot N-P=0.$$

The structural resistance (friction force) *R*_u_ and the load effect *R*_n_ both comply with the normal distribution. Based on the first-order second-moment, the following equation is obtained to predict the reliability index *β*:

(30) $$\beta =\frac{\ln(R_{m}/Q_{m})}{\sqrt{V_{R}^{2}+V_{Q}^{2}}}=\frac{\ln K_{m}}{\sqrt{V_{R}^{2}+V_{Q}^{2}}},$$

where *R*_m_ is the average value of the structural resistance *R*_u_, *Q*_m_ is the average value of the load effect *R*_n_, *V*_R_ is the coefficient of variation of the structural resistance *R*_u_, and *V*_Q_ is the coefficient of variation of the load effect *R*_n_. *V*_R_ is set to 0.122 according to the experimental statistical parameters and *V*_Q_ is set to 0.050. *K*_m_ is the average value of the ratio of the structural resistance *R*_u_ to the load effect *R*_n_.

The calculated reliability indices of the design formulae are listed in Table 4, where the test results and the bearing capacity calculated by the four design formulae are used as the structural resistance *R*_u_ and the load effect *R*_n_, respectively. As can be seen from Table 4, the reliability index *β* predicted by the 50% *G*_t_-*F*_f_-*N* design formula is small, indicating that the predictions are unconservative. The reliability index *β* calculated by the 99.9% *G*_t_-*F*_f_-*N* design formula is 3.09 (the largest), which illustrates that the predictions tend to be conservative. Moreover, the reliability indexes predicted by the 95% *G*_t_-*F*_f_-*N* design formula and the fitting design formula are about 1. Overall, the different guaranteed rates can reflect the security degree of the design formulae well.

### 7.2. Resistance Partial Coefficient

The reliability checking equation of the structure can be expressed as:

(31) $$R_{u}\leq R_{n}/\gamma_{R},$$

where *γ*_R_ is the partial coefficient of the structural resistance *R*_u_.

Based on the first-order second-moment, the reliability index *β*is determined by:

(32) $$\beta =\frac{\ln(\gamma_{R}R_{m}/Q_{m})}{\sqrt{V_{R}^{2}+V_{Q}^{2}}}=\frac{\ln(\gamma_{R}K_{m})}{\sqrt{V_{R}^{2}+V_{Q}^{2}}}.$$

According to Equation (32), Equations (33)–(36) are derived to calculate the partial coefficients of structural resistance *γ*_R_ of design formulae.

For the partial coefficients of structural resistance *γ*_R_ of the 50% *G*_t_-*F*_f_-*N* design formula:

(33) $$\gamma_{R}=e^{\left(0.1245\beta -0.009\right)}.$$

For the partial coefficients of structural resistance *γ*_R_ of the 95% *G*_t_-*F*_f_-*N* design formula:

(34) $$\gamma_{R}=e^{\left(0.1245\beta -0.190\right)}.$$

For the partial coefficients of structural resistance *γ*_R_ of the 99.9% *G*_t_-*F*_f_-*N* design formula:

(35) $$\gamma_{R}=e^{\left(0.1245\beta -0.407\right)}.$$

For the partial coefficients of structural resistance *γ*_R_ of the fitting design formula:

(36) $$\gamma_{R}=e^{\left(0.1245\beta -0.128\right)}.$$

**Figure 22:**
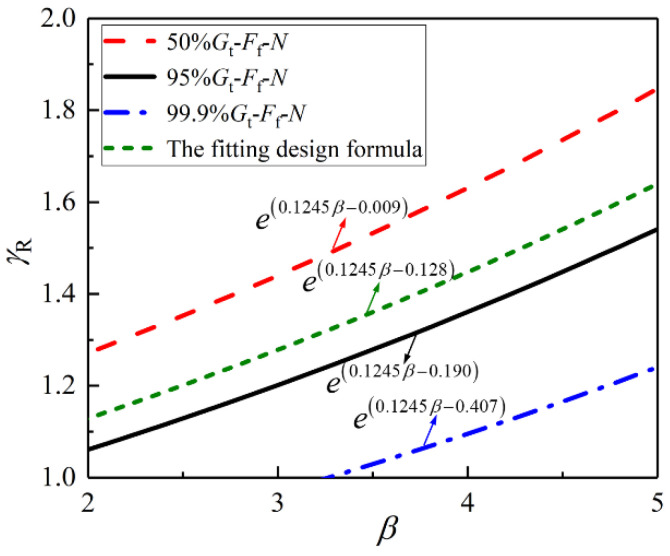
*γ*_R_-*β* curves.

As can be seen from Figure 22, the partial coefficient of structural resistance *γ*_R_ increases with the increase of the reliability index *β,* and the relationship was approximately linear. Moreover, the *γ*_R_-*β* curve (50% *G*_t_-*F*_f_-*N*) is higher than the other curves, indicating that, under the same reliability index *β*, the *γ*_R_ of 50% *G*_t_-*F*_f_-*N* formula is the largest, which is due to the design formula tending to be unconservative without considering the *γ*_R_. The *γ*_R_-*β* curve (95% *G*_t_-*F*_f_-*N*) and the *γ*_R_-*β* curve (the fitting design formula) are close to each other and are located between the 50% *G*_t_-*F*_f_-*N* curve and the 99.9% *G*_t_-*F*_f_-*N* curve.

## 8. Conclusions

Thirty-six specimens were tested to investigate the frictional properties of the composite interface between the raw bamboo and the steel hoop (BHC), to find proper methods for calculating the sliding friction force, and to provide reasonable design details for the BHC. Based on the experimental and theoretical study, the major conclusions can be drawn:

- The failure of the specimens showed a certain ductility. The experimental results mainly showed two failure modes of all specimens: continuous longitudinal slip after the vertical load reaches the peak; the steel hoop is stuck in the bamboo skin after a period of slip.
- Based on probability analysis, the interfacial friction coefficient for BHC under three guaranteed rates (50%, 95%, and 99.9%) are 0.115, 0.096, and 0.077, respectively, which can reflect the security degree of the connection, respectively. By regression analysis, the fourth interfacial friction coefficient is 0.103, equivalent to the 81.94% guaranteed rate.
- This type of bamboo connection can be used in the spatial structure of raw bamboo, as shown in Figure 2. The pretension value of the bolt can be determined according to the compression strength perpendicular to grain of bamboo, and the diameter and grade of bolts can then be determined. Therefore, based on the pretension value of the bolt, the maximum friction provided by a single connection can be calculated. The number of steel hoops can be configured according to the design load in practical engineering applications. It is suggested to use a digital electronic torque wrench, by which the torque corresponding to the pretension is applied.
- The following design details are suggested: the raw bamboo should be used with the circular cross-section, flat surface, and consistent diameter along the length; if the diameter of bamboo is inconsistent, the smaller diameter end of the vertically placed bamboo should be placed above; as load-bearing bamboos, the bamboo age should not be less than 4 years, and the moisture content is generally between 8% and 12% [14].
- The reliability indices of the four design formulae are up to 0.07, 1.44, 3.09, and 0.97, respectively, and resistance partial coefficients are suggested accordingly.

## Figures and Tables

**Table 1:** Parameters and the peak load of specimens.

Specimens	*N* (kN)	*P*_c_ (kN)	*μ*	Stiffness (kN/mm)	Ductility Cofficient	*P*_u_ (kN)	*P*_FEM_ (kN)
PO-1-1~PO-1-13	1.84 (200 με)	0.29	0.120	0.14	2.63	0.222	0.201
PO-2-1	1.38 (150 με)	0.22	0.125	0.13	3.13	0.172	0.165
PO-2-2, PO-2-3	2.76 (300 με)	0.44	0.150	0.21	2.97	0.415	0.400
PO-2-4, PO-2-5	4.14 (450 με)	0.66	0.130	0.20	2.16	0.538	0.526
PO-2-6, PO-2-7	4.6 (500 με)	0.73	0.090	0.19	2.75	0.419	0.408
PO-2-8~PO-2-10	5.52 (600 με)	0.88	0.103	0.24	3.03	0.571	0.561
PO-2-11, PO-2-12	6.9 (750 με)	1.10	0.113	0.23	2.40	0.775	0.764
PO-2-13	7.36 (800 με)	1.17	0.083	0.26	4.58	0.611	0.601
PO-2-14, PO-2-15	8.28 (900 με)	1.32	0.095	0.28	2.23	0.784	0.768
PO-3-1, PO-3-2	2.072 (225 με)	0.33	0.145	0.15	2.00	0.299	0.281
PO-3-3	2.76 (300 με)	0.44	0.160	0.19	4.02	0.443	0.420
PO-3-4	4.14 (450 με)	0.66	0.085	0.17	3.92	0.357	0.348
PO-3-5	5.52 (600 με)	0.88	0.075	0.21	3.46	0.408	0.392
PO-3-6	6.9 (750 με)	1.10	0.093	0.21	1.68	0.646	0.634
PO-3-7	8.28 (900 με)	1.32	0.113	0.23	2.64	0.933	0.914
PO-3-8	9.66 (1050 με)	1.54	0.073	0.25	1.78	0.702	0.688

Note: *N* is the contact pressure, that is, the sum of contact stress on the inner surface area of the steel hoop; *P*_c_ is the pretension force of a high-strength bolt; Ductility coefficient calculated by Δ_u_/Δ_y_ is used to evaluate the deformability of the connections, where Δ_u_ and Δ_y_ are the ultimate and yield displacement, respectively. *P*_u_ and *P*_FEM_ is the average value of the bearing capacity of the duplicate specimens predicted by the test and FE model, respectively; *μ* is the friction coefficient obtained by dividing the friction force by the contact pressure.

**Table 2:** Measured material properties.

Steel				Raw Bamboo				
*f*_y_ (MPa)	*E*_s_ (MPa)	Poisson’s Ratio *v*	*f*_u_ (MPa)	Moisture Content	Tangential Shrink Ratio	Radial Shrink Ratio	Water Absorption Rate	Compressive Strength Parallel to Grain (MPa)
276.34	1.98 × 10^5^	0.29	334.42	12.32	2.18	2.59	75.85	50.90

**Table 3:** Summary of the test data for other types of bamboo connections.

Sources	Type of Connections	Specimens	*D* (mm)	Screw Type	*P*_u_ (kN)
[5]	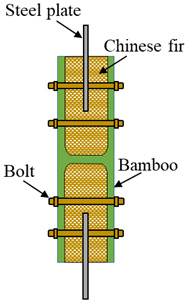	WS-8-50	99.28	M8	5.13
		WS-10-50	105.83	M10	12.57
		WS-12-50	101.87	M12	17.58
		WS-14-50	93.89	M14	14.79
		WS-16-50	107.73	M16	15.06
		WS-8-80	101.57	M8	5.88
		WS-10-80	101.15	M10	11.24
		WS-12-80	97.62	M12	15.65
		WS-14-80	82.75	M14	21.01
		WS-16-80	86.35	M16	24.24
		WS-8-110	88.97	M8	5.77
		WS-10-110	99.99	M10	11.18
		WS-12-110	84.84	M12	14.42
		WS-14-110	95.26	M14	20.26
		WS-16-110	81.12	M16	20.50

**Table 4:** Calculation results of reliability indices of the design formulae.

Design Formulae	*V*_R_	*V*_S_	*K*_m_	*β*
50% *G*_t_-*F*_f_-*N*	0.122	0.050	1.01	0.07
95% *G*_t_-*F*_f_-*N*			1.21	1.44
99.9% *G*_t_-*F*_f_-*N*			1.50	3.09
The fitting formula			1.14	0.97

## Data Availability

Data is contained within the article.
